# Biomechanics of the lead straight punch and related indexes between sanda fighters and boxers from the perspective of cross-border talent transfer

**DOI:** 10.3389/fphys.2022.1099682

**Published:** 2023-01-16

**Authors:** Yang Liu, Lei Li, Xianliang Yan, Xinseng He, Bin Zhao

**Affiliations:** ^1^ Sports Science Postdoctoral Research Station, Chengdu Sport University, Chengdu, China; ^2^ School of Physical Education, Nanchang Normal University, Nanchang, China; ^3^ Chinese Guoshu Academy, Chengdu Sport University, Chengdu, China; ^4^ School of Wushu, Chengdu Sport University, Chengdu, China; ^5^ School of Physical Education, Nanchang Institute of Technology, Nanchang, China

**Keywords:** sanda, boxing, athlete, lead straight punch, striking effect, legs, index

## Abstract

**Objective:** To bridge the technical gaps in reserve athletes in China’s national boxing program and to rapidly improve the overall level of boxing, the Wushu Sports Administration Center of the General Administration of Sports of China has sent outstanding Sanda players to boxing events through cross-border talent transfer. This was done to widely improve the strengths and resources in various fields to prepare for the Tokyo Olympic Games. In view of this, we analyzed and compared differences in biomechanical parameters of the lead straight punch and index of force developments of the lower extremities between Sanda and boxing. The results provide information and insights to bridge the technical gaps in cross-border talent transfer from Sanda to boxing.

**Methods:** We employed a Vicon infrared 3D motion capture system, two Kistler platforms, a Kistler target, and a synchronous instrument. Eleven boxers and sixteen Sanda athletes were recruited, and their lead straight punch techniques were compared and analyzed. Three indexes of punch velocity, six indexes of strength measurement, and four indexes of lower extremity strength were analyzed.

**Results:** Significant differences in the peak punch velocity and contact velocity were found between the two groups. Furthermore, significant differences were noted in the peak impulse, peak strength, relative strength, and the rate of force development (RFD). Among the kinetics indexes of lower limbs, the front leg strength index was higher in the boxing group than in the Sanda group, namely the RFD index and RFD/body mass.

**Conclusion:** Based on the disparity in the effects of the lead straight punch and biomechanical parameters of both lower extremities, we can conclude that, compared to the boxers, most Sanda athletes lack standard punching technique. Therefore, it is advised that coaches and practitioners carefully consider selecting Sanda athletes with higher technical standards of punching.

## 1 Introduction

Athlete selection and training of athletes are important components of success in international competitive sports (Pinder et al., 2013), and a large number of athletes achieve rapid improvement of their competitive level in new sports through cross-border talent transfer (Collins et al., 2014; MacNamara and Collins, 2015; Saether et al., 2021). Against the backdrop of the reform of the competitive sports system and the intense Olympic preparation, China is committed to efficiently train athletes with high technical and tactical level for the Olympic Games through the implementation of cross-sport talent transfer. On the other hand, the cross-sport talent transfer can also further improve the selection and training system of our existing reserve talents. Cross-sport and cross-event talent transfer refers to selecting athletes from other sports such as Sanda and Wushu who have already reached a certain training or competition level and have the innate ability or experience required for the new sport in an attempt to train athletes to achieve the world level quickly. It also provides an organized selection and training process for these athletes. In order to make up for a shortage of reserve talent in China’s boxing program and to rapidly improve the overall competition level of boxing, the Wushu Sports Management Center of the State General Administration of Sports filled the boxing program with outstanding Sanda athletes by means of cross-sport talent transfer to extensively mobilize the strengths and resources of all fields in preparation of the 2020 Olympic Games in Tokyo, Japan.

Combat sports such as boxing, martial arts, Sanda, taekwondo, and mixed martial arts have certain similarities in fighting techniques, such as striking. Boxing is an Olympic sport, and many athletes from combat sports choose to join national boxing teams through cross-sport talent transfer. However, due to limitations in competition rules, athletes in the same weight class will require targeted individualized training according to their own characteristics and the requirements of the sport, which creates barriers for cross-border talent transfer. To aid in overcoming these challenges, this study intended to objectively and accurately evaluate differences in biomechanical indexes of the key biomechanical aspects of the lead straight punch between boxing and Sanda and, thus, provide guidance and comprehensive training for cross-sport talent transfers for combat sports. The straight punch is one of the most basic techniques in boxing. Joseph, a famous American trainer, stated that mastering the straight punch means mastering 80% of boxing techniques. The straight punch is divided into a lead straight punch and a rear straight punch. The lead straight punch features short distance, fast speed, short time, and good effect. The lead straight punch technique can deliver powerful blows, but can also be combined with light or heavy strikes to interfere with opponents. In recent years, an increasing number of kinematic and dynamic indexes have been measured for direct punching techniques, mainly focusing on punching speed, power, punching time, and punching effect (Smith et al., 2000; Stojsih et al., 2010). For example, Walilko et al. used a Hybrid III dummy with an integrated accelerometer and other intelligent equipment to study the facial force of straight punches and the accelerated velocity of fascial rotation to judge the risk of concussion (Walilko et al., 2005). Some researchers have used infrared motion capture systems to study indexes of boxing athletes’ peak punch speed and contact punch speed (Piorkowski et al., 2011). Some researchers have also used this platform to test the peak strength of striking (Lenetsky et al., 2013b; Loturco et al., 2016). In boxing, striking power comes from the lower limbs (Turner et al., 2011), and, to some extent, related indexes of lower limbs affect the straight punch (Rimkus et al., 2019). The ratios of maximum strength to body weight of the lead and rear feet and of speed force index to body mass were significantly positively correlated with punching speed (Su et al., 2013). Therefore, relevant kinematic and dynamic variables provide reference points to explore the effects of straight punches in Sanda athletes and boxers.

## 2 Materials and methods

### 2.1 Participants

The lead straight punch technique was the main research object of this study. There were 11 boxers, including 3 nation lever athletes, 4 athletes at level I, and 4 athletes at level II. There were 16 Sanda athletes, of which 2 were at the master level, 10 athletes were level I, and 4 athletes were level II. The participants were tested according to S1 to S27 numbers, and the participants were divided into a boxing group [height (mean ± standard deviation) 175.25 ± 7.81 cm; body mass, 66.06 ± 9.76 kg] and a Sanda group (height, 175.57 ± 7.18 cm; body mass, 68.71 ± 9.51 kg). There were no significant differences in height and body weight between the two groups (t = 0.927, t = 0.552; *p* > 0.05). In order to accurately control comparisons, the athletes were all from the same “weight class”. Before testing, basic indexes (height, weight, etc.) were recorded, and there were no significant differences between the groups.

### 2.2 Protocol

This study was reviewed by professors and experts from the School of Sports Science, Shanghai University of Sport (Shanghai, China). Experiments were carried out in strict accordance with the Helsinki Declaration and were approved by the Ethics Committee of the Shanghai University of Sport. Experiments were performed in the experimental hall of the School of Sports Science, Shanghai University of Sport, at a temperature of 25°C ± 1°C. Before entering the test hall, participants were familiarized with the experimental protocol and any potential risks. Prior to physical fitness testing, a standardized warm-up protocol (i.e., 15 min of dynamic stretching, running) was performed. The test platform was fixed on a tripod which that was fully covered by a thin layer of foam to prevent impact injuries to the boxers, who used competition gloves to perform striking tests. The hand point (right or left: orthodox or southpaw stance), which was labelled on the central position of the back of the fist with double-sided adhesive, was used to track the position of the fist and to calculate its velocity. During testing, in order to properly adjust the distance of the force target, participants stood on two embedded force platforms (90 cm × 60 cm, built-in signal amplifier, 1,000 Hz; 9287 B; Kistler Group, Winterthur, Switzerland) in the preparation position and struck the target with full force using a straight punch. Three effective punches were tested per athlete, and the best straight punch performance was taken for analysis.

### 2.3 Instruments and equipment

**FIGURE 1:**
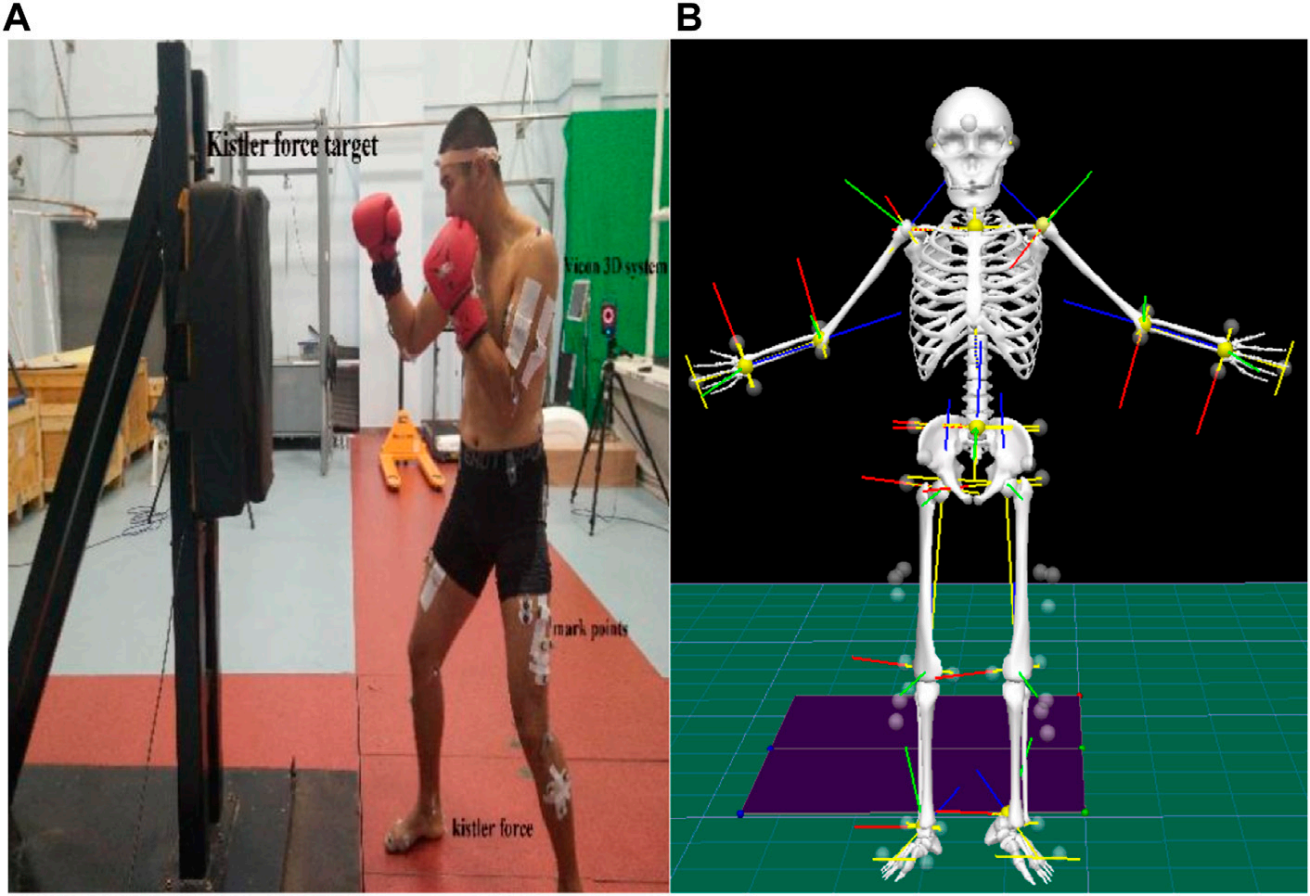
**(A)** boxer’s preparation posture with Kistler’s strength target fixed on the tripod; **(B)** corresponding rigid-body model.

The study used one force target (1,000 Hz; Kistler Group) fixed on a tripod to record the strength of strikes and two embedded force platforms to record changes in leg forces. A motion capture system (Vicon Nexus, 16 cameras; 200 Hz; software v2.6.1; Vicon Motion Systems, Ltd., Oxford, United Kingdom) and reflective marking balls were used to mark individual joints for modeling. The motion capture system was used to record kinematic data of the participants, calculate displacement, and derive the velocity. The digital signals collected by the motion capture system and the force platforms were converted into synchronized analogue signals using a synchronization device (Figure 1).

### 2.4 Index selection and data processing

#### 2.4.1 Index selection

##### 2.4.1.1 Force plate for punching index

**FIGURE 2:**
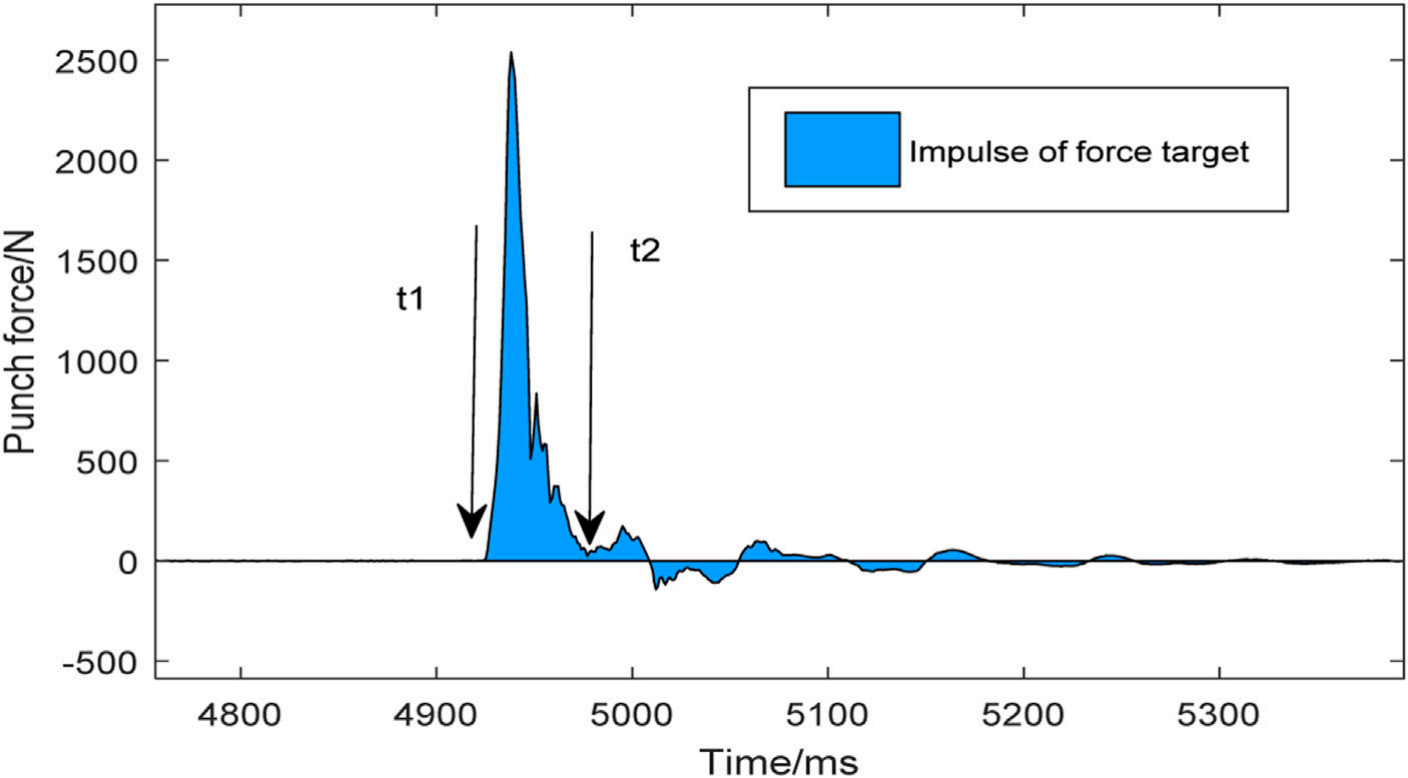
Impulse of a straight punch.

The impact impulse is defined as the area under the curve of the force and the time when the fist contacts and leaves the target. Mathematically, this is the integral of force *F(t)* with respect to time (Figure 2: t1–t2). The function of the impact impulse is shown in Eq. 1.

$$I=\int_{t1}^{t2}F\left(t\right)\;dt$$ (1)

The peak force is defined as the force value curve interaction time action to the peak when the fist contacts the force measuring target. The impact force rate is defined as the linear data peak force of the force-measuring target divided by the time to reach the peak force.

##### 2.4.1.2 Ground Reaction Force Index

Three-dimensional ground reaction force data were derived from the embedded force platforms. Participants stood with both legs on the embedded force platforms to record changes in start-up strength of both legs. The strength index is defined as the force rate divided by body mass. The rate of force development (RFD) is the peak force/peak time (*F*_*max*_/*T*_*max*_) (Suchomel et al., 2014) (Aeles et al., 2022). RFD also defines the lower-limb indexes of start-up strength, namely RFD/body mass, peak force, and the time of peak force.

##### 2.4.1.3 Punching Velocity Index

The peak punch velocity, contact velocity, and deceleration rate were calculated. The peak punch velocity is the peak point on the velocity time-series after the marked point displacement of the marked punch is time-derivative. The calculation of contact velocity can be modeled using the motion capture software, where the previous frame was synchronized when the first contact the Kistler force target. The velocity is obtained by taking the displacement derivative of the marked point (hand), and the acceleration can be obtained by taking the derivative of the velocity. The index calculation method of the deceleration rate is as follows:

$$Deceleration\;rate=\frac{Peak\;velocity-Contact\;velocity}{Peak\;velocity}$$ (2)

#### 2.4.2 Data processing and V3D modeling

This study used Vicon Nexus software to interpolate missing mark points, and the data after dot filling were imported into V3D software for human modeling (Stanley et al., 2018) (Figure 1B) and kinematic and dynamic analysis. The “start event” and “finish event” tags were defined to obtain the export kinematics and GRF data in ASCII format for further analysis in Microsoft Excel (Microsoft, Redmond, WA, United States). Z-axis (vertical data) punching force data from the force targets were analyzed using Bioware software. Analysis of lower limb data used three axes to determine the combined force, as shown in Eq. 3.

$$F=\sqrt{x^{2}+y^{2}+z^{2}}$$ (3)

#### 2.4.3 Statistical analysis

Relevant data were imported into SPSS (v24.0; IBM Corp. Armonk, NY, United States) for analysis, and the single-sample Kolmogorov–Smirnov test was used to determine normality. A parametric test was used for normally distributed data and a non-parametric test was used for non-normally distributed data. Data are represented herein as mean ± standard deviation. The independent sample t-test was used to compare differences in various indexes of the lead straight punch of athletes of different levels. The significance level was set at *p* < 0.05. The standardized effect size (Cohen’s d) was used to interpret the magnitudes of differences between the lead straight punching data. A common interpretation of effect sizes is: small (d = 0.2), medium (d = 0.5), and large (d = 0.8).

## 3 Results

### 3.1 Force target data

**FIGURE 3:**
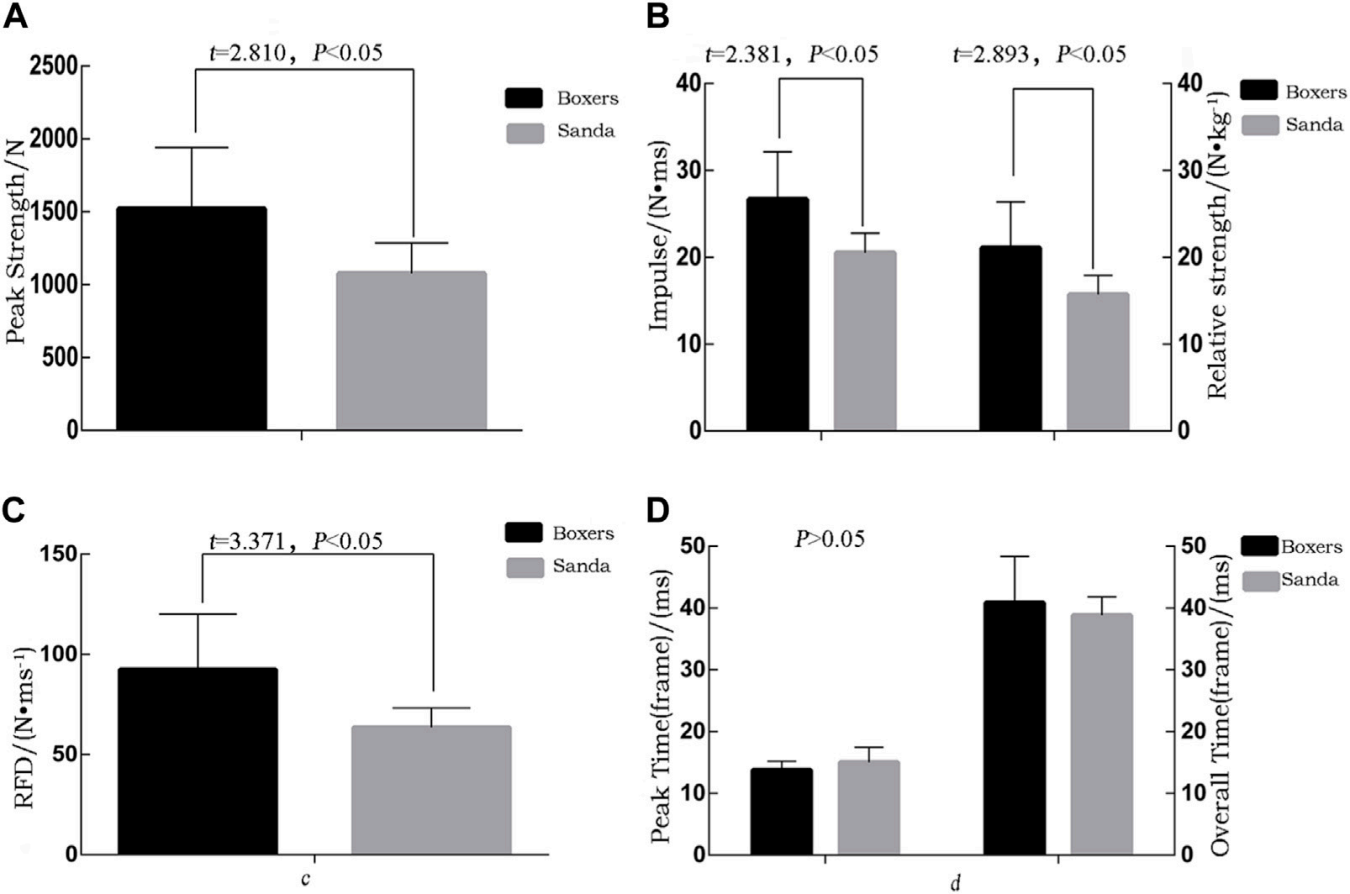
**(A)** peak strength of straight punches for boxing and Sanda; **(B)** relative strength of the impulse; **(C)** peak force/time; **(D)** force and time curve of the target.

Six secondary indexes, namely the punch impulse, peak strength, relative strength, peak (frame) time, RFD, and movement time, were selected among the primary indexes from the force targets from lead straight punches. Four indexes showed statistically significant differences between the boxing and Sanda groups.The boxing group, compared to the Sanda group, had higher in the peak impulse (26.66 ± 5.49 N•ms vs. 20.53 ± 2.23 N•ms, *Cohen’s d* = 1.46, *p* < 0.05), in peak strength [(1,520.80 ± 420) N vs. (1,077 ± 209) N, *Cohen’s d* = 1.34, *p* < 0.5], in relative strength (21.08 ± 5.30) N•kg^−1^ vs. (15.69 ± 2.23) N•kg^−1^, *Cohen’s d* = 1.33, *p* < 0.05), in RFD (92.47 ± 27.72 N•ms^−1^ and 63.55 ± 9.81 N•ms^−1^, *Cohen’s d* = 1.39, *p* < 0.05).The other two indexes were not significantly different between the boxing and Sanda groups, namely full overall time [(43.57 ± 3.86) ms vs. (43.10 ± 3.38) ms, *t* = 0.26, *p* > 0.05] and peak time (16.51 ± 1.13) ms and (17.00 ± 2.53) ms, *t* = −0.414, *p* > 0.05; Figure 3).

### 3.2 Punch velocity index

**FIGURE 4:**
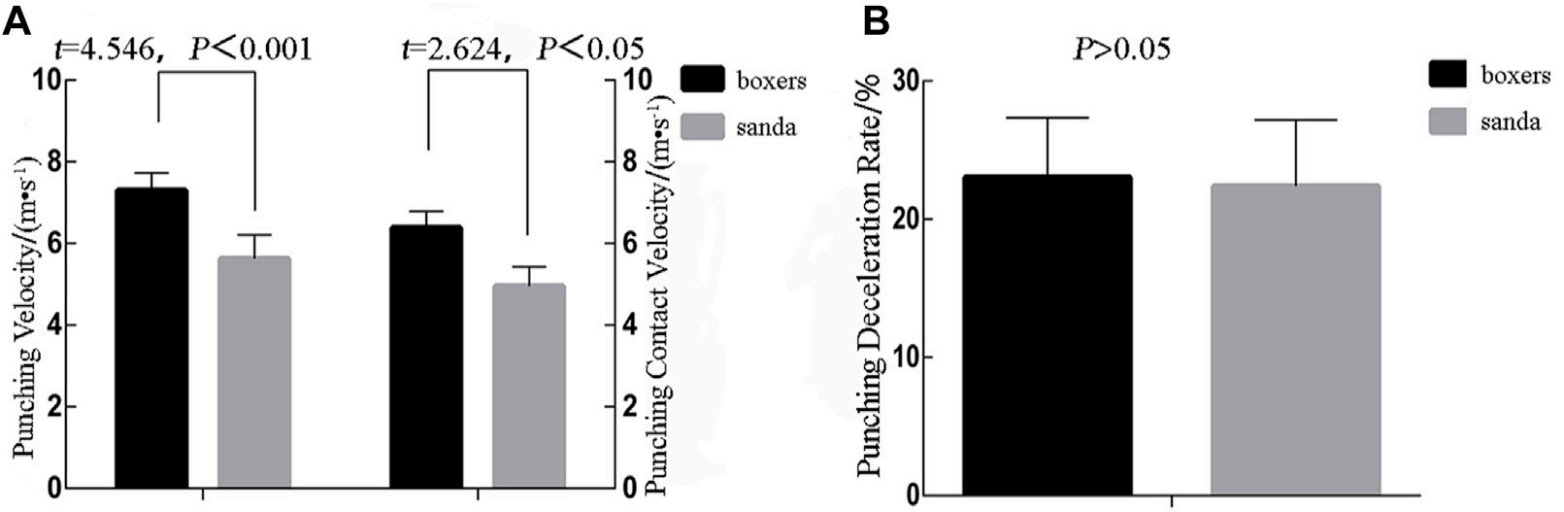
**(A)** punching velocity and contact velocity of straight punches for boxing and Sanda; **(B)** percentage decay of punching velocity.

The peak punch velocity index of the lead straight punch showed significant differences between the boxing and Sanda groups (7.31 ± 0.42 vs. 6.38 ± 0.41 m•s^−1^, *t* = 4.546, *Cohen’s d* = 2.23, *p* < 0.001); contact punch velocity (5.63 ± 0.584 vs. 4.95 ± 0.47 m•s^−1^, *t* = 2.624, *Cohen’s d* = 1.22, *p* < 0.05). In terms of the velocity decay rate, the decay rate fluctuation amplitude of the boxing group was not significantly different from that of the Sanda group (23.05 ± 4.3 vs. 22.38 ± 4.8, *t* = 0.295, *p* > 0.05). (Figure 4).

### 3.3 Bilateral lower limb strength indexes

**FIGURE 5:**
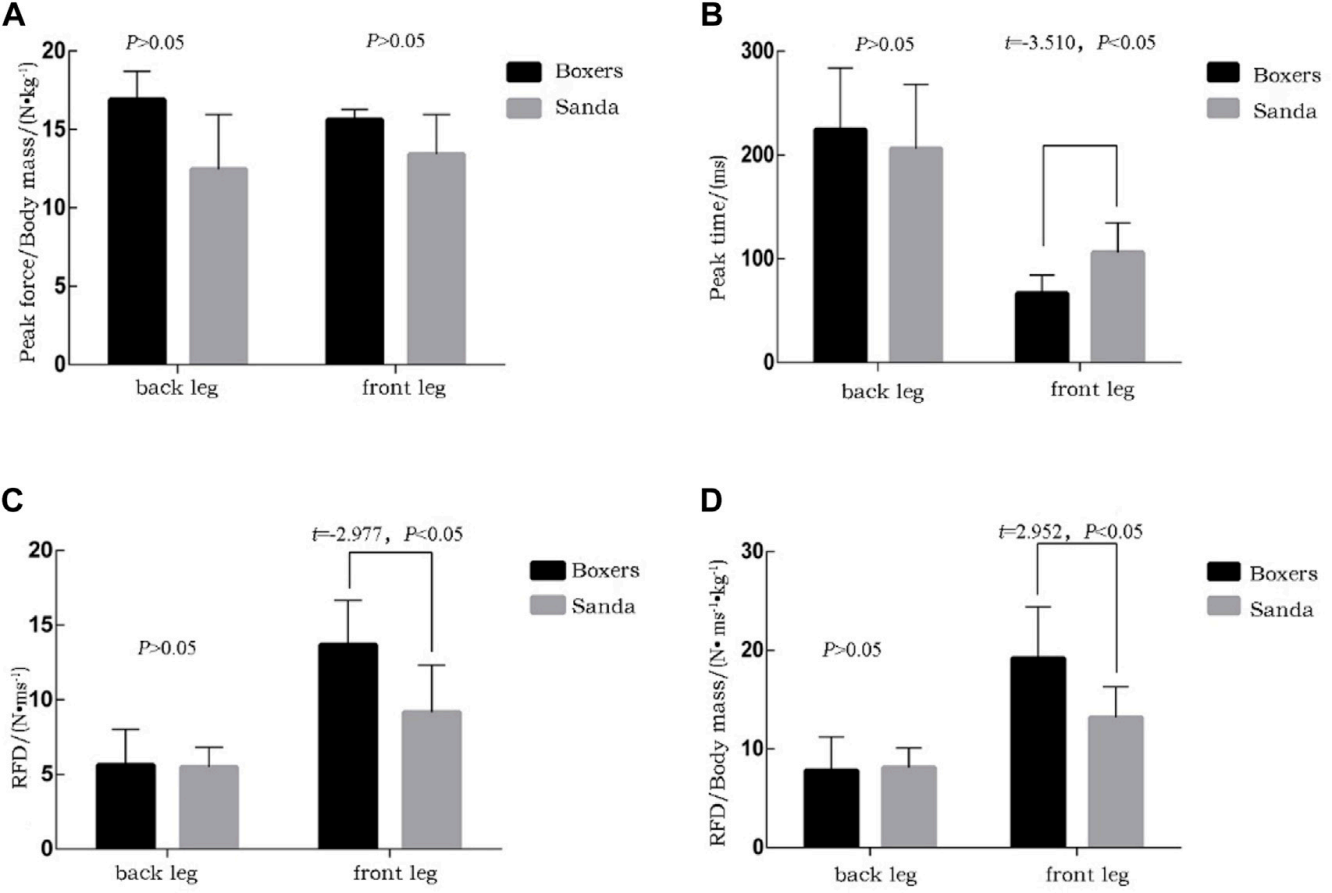
**(A)** peak lower limb force divided by body weight for boxers and Sanda athletes; **(B)** time to peak force in lower limbs; **(C)** athletes’ lower limb rate of force development and **(D)** relative rate of force development.

Five indexes were calculated from the data from the lower limb dynamometers (start-up strength): peak strength, peak strength/body mass, time of peak force, RFD index, and RFD/body mass. The five front-leg start-up strength indexes differed statistically between the boxing and Sanda groups. The boxing group, compared to the Sanda group, had higher peak strength/body mass (12.46 ± 3.48 N•kg^−1^ vs. 13.42 ± 2.53 N•kg^−1^, *p* > 0.05), RFD index (13.67 ± 2.98 N•ms^−1^ vs. 9.17 ± 3.13 N•ms^−1^, *t* = 2.977, *Cohen’s d* = 1.48, *p* < 0.05), RFD/body mass (19.18 ± 5.2% N•ms^−1^kg vs. 13.2 ± 3.1% N•ms^−1^•kg^−1^, *Cohen’s d* = 2.33, *p* < 0.05). The boxing group showed shorter time of peak force than the Sanda group (198.75 ± 33.34 ms vs. 301.43 ± 93 ms, *Cohen’s d* = −1.47, *p* < 0.05). Among the five start-up strength indexes for the back legs, three were not statistically different between the two groups, namely peak strength/body mass (16.909 ± 1.801 N•kg^−1^ vs. 15.61 ± 0.65 N•kg^−1^, *t* = 1.811, *p* > 0.05), RFD/body mass (7.8 ± 3.4% N•ms^−1^•kg^−1^ vs. 8.1 ± 2.0% N•ms^−1^•kg^−1^, *p* > 0.05), and back leg time to peak (224.29 ± 59.68 ms vs. 206.00 ± 62.21 ms, *p* > 0.05). Full time is no significantly difference (432.86 ± 62.90 ms vs. 439.00 ± 50.87 ms, *p* > 0.05). Front leg time of peak force is significantly difference (66.71 ± 17.71 ms vs. 106.00 ± 28.63 ms, *t* = −3.510, *Cohen’s d* = −1.65, *p* < 0.05). RFD index is also significantly difference (5.63 ± 2.39 N•ms^−1^ vs. 5.49 ± 1.34 N•ms^−1^, *Cohen’s d* = −1.47, *p* < 0.05). Overall, the RFD index of the front leg differed significantly between the boxing and Sanda groups, and the differences in the strength of the front leg were only shown in individual indexes (Figure 5).

## 4 Discussion

### 4.1 Punching strength indexes compared between sanda and boxing athletes

The peak strength of boxing and Sanda groups differed significantly from studies from other countries (P Bolander et al., 2009; Vaslin, 2005) (Buśko et al., 2016; Halperin et al., 2016) (T. J. Walilko et al., 2005) (Smith et al., 2000) (S Lenetsky et al., 2018). In addition, the peak strength/body mass ratio was used to obtain the relative punch strength (Dunn et al., 2019). The relative strength of the boxing group was 21.08 ± 5.30 N•kg^−1^, which was significantly higher than the relative strength of the Sanda group (15.69 ± 2.23 N•kg^−1^) and significantly lower than the relative strength of the boxers reported by Krzysztof Buśko et al. (19.19 ± 5.02 N•kg^−1^) (Buśko Krzysztof, 2014).

Power indexes in boxing can reflect the effects of punching (i.e., whose punches are “heavy”), especially in the framework of newer rules for boxing, which require “clear, powerful, and unobstructed” striking. Over the course of a boxing match, whoever’s fist is “heavy” has the advantage. Regardless of the level, the “heavier” a boxer punches their opponent, the more damage it will cause.

The present study found a significant difference between boxing and Sanda athletes in punching effect, with boxing group showing significantly higher effect than the Sanda group. The punch impulse and RFD were significantly higher in the boxing group. Moreover, the peak power and relative power indexes of lead straight punching for athletes of the same weight class were significantly higher in the boxing group than the Sanda group, indicating that Sanda athletes do not throw lead straight punches as strongly. This may be related to the competition rules of Sanda and the athletes’ own characteristics, as they may focus more on holding and whipping. Regarding technical requirements, Sanda athletes are not only required to hit the upper limbs powerfully but also require the ability to kick and hold the lower limbs with their fists and legs. This causes Sanda athletes to punch irregularly. If they are changing their sport to boxing, they should focus on improving their punching techniques and striking effects. On the other hand, “wrestling” (a technique that mainly relies on the strength of the arms to bring the opponent down) is better than “punching” in close combat. According to the rules of boxing competition, the opponent must be hit by a punch and show obvious displacement to earn a point. It is far more beneficial for a Sanda athlete to use the wrestling technique in close combat than to throw punches, as two points are awarded for effective wrestling in close combat. For Sanda athletes in close combat, long distance techniques are usually used, such as the “leg whip” (a general term for the curved leg technique in Sanda), “side kick” (a straight-line offensive technique), and other technical movements. The tactical style of “long-range kicks and close-range grappling” is also often used in Sanda matches. Therefore, differences in the rules of the sports directly affect the differences in training strategies. Sanda athletes do not throw standard punches. From the perspective of cross-sport talent transfer, it is not difficult to change the coordination of upper and lower limbs and the punching effects of Sanda athletes, as long as accurate measurements are taken and players with good lead straight punching effects are selected. As both Sanda and boxing are combat sports, cross-event talent transfer between them is possible.

In conclusion, considering differences in striking effectiveness in boxing of Sanda athletes, one should accurately measure and screen athletes with relatively good punching techniques, such as straight lead velocity and continuous punching velocity, when training talent transfer athletes. In addition, during the bridging training phase after the crossover, the quality of effective striking should be targeted to improve the momentum of punches and improve the striking effect.

### 4.2 Comparing punching velocity indexes between sanda and boxing athletes

As boxing rules have changed, the lead straight punch has become more important, and studies have shown that it is the most frequently used technique. For example, cross-event athlete Gu Hong is skilled in using the lead straight punch; during the Tokyo Olympics in Japan, she heavily utilized the lead straight punch—which has the advantages of being fast, accurate, and firm—by continuously throwing lead straight punches to interfere with her opponent, thus ensuring success. She made it all the way to the final, and was the runner-up in the women’s 69 kg weight class. The present study showed that the peak velocity and contact velocity of lead straight punches of boxing athletes were faster than those of Sanda athletes, also with higher impulse values. In addition, the contact velocity index was also influenced by the peak velocity, and the most successful boxers showed higher peak velocity. Moreover, the strength measurement target F(t)-t curve (i.e., the hitting strength) was higher in the boxing group in terms of strength time. In terms of time indexes, there was no significant difference between groups. The peak velocity and contact velocity in boxing determine the effect of striking with the lead straight punch, and it is necessary to focus on improving these parameters for cross-event talent transfer. Therefore, Sanda athletes who switching to boxing should perform strength resistance training such as upper body complex resistance training, ballistic resistance training, and fast stretch resistance training.

In summary, the peak velocity and contact velocity indexes of the lead straight punch are key indexes for evaluating the striking effectiveness of both boxing and Sanda athletes. Therefore, in view of the disadvantage seen in punching velocity in Sanda athletes, athletes with better punching velocity or continuous punching velocity should be selected for cross-event talent transfer.

### 4.3 Differences in the start-up ability of lower limbs between sanda and boxing athletes

The strength of striking comes from the lower limbs (Lenetsky et al., 2019), as power is transmitted from the feet and legs upward. Lower limb CMJ (countermovement jump) index of boxers is positively correlated with the power of punching (Pilewska et al., 2017) with a correlation coefficient of 0.67–0.85 (Loturco et al., 2015). There is also significant positive correlation between the maximum punching velocity of the lead straight punch and the maximum horizontal ground strength of the back leg (*y*-axis). The lower extremities (Turner et al., 2011) play an important role in the upper limb striking process and in lead straight punching technique. In the present study, there was a significant difference in the indexes of the front leg dynamometer strength between boxing and Sanda athletes, while there was no significant difference in the indexes of the back leg dynamometer. There was also no significant effect of the indexes of the back leg active strength on lead straight punching technique in the two groups. Compared with the rear straight punch, which has “front leg support and back leg generate force”, front leg strength plays a very important role in lead straight punching.

There was a significant difference in the time to peak strength (start-up strength time) of the front leg in the two groups (Figure 5B). This also represents a shorter active strength time of the foreleg for the lead straight punch in the boxing group and a longer start-up strength time of the foreleg in the Sanda group, while the start-up strength of the front leg contributed more to the lead straight punch. In Sanda, the longer start-up time of the front leg is not conducive to the swiftness of the lead straight punching technique. Therefore, if a Sanda athlete transfers to boxing, they should focus their technical training on shortening the front leg start-up time to create a strong striking effect.

The RFD index is the ratio of peak strength to time, which is an evaluation index of power (Z.P., 2013). Zhang and Liu (2008) found that the correlation between the instantaneous RFD and off-ground velocity of excellent high jumpers was as high as 0.965 (Zhang and Liu 2008). In boxing, Su et al., 2013a, used two force platforms to investigate correlations between the back leg force index and punching velocity, which was as high as 0.882 (Su et al., 2013a). The authors proposed that increasing the maximum strength of the start-up phase of both legs could help to improve punching velocity. The maximum peak strength/body mass ratio of both legs and the rapid strength index/body mass ratio were positively correlated with punch velocity. Shortening the power generation time and fast power generation can increase punching velocity (Su et al., 2013a). The present study illustrated that the indexes of start-up generate force of the front leg are the most important indexes to improve the velocity and power of the lead straight punch, while the general index of the foreleg power of Sanda athletes was lower than that of the boxing group. It further illustrated that the start-up power of the lower limbs (front leg) in lead straight punching in Sanda athletes should be further standardised technology. Improving the coordinated power generation ability of upper and lower limb punching is an important responsibility of coaches. Furthermore, special attention should be paid to evaluating players with better lower limb abilities when transferring athletes across disciplines (Lenetsky et al., 2013a).

In conclusion, given the significant differences in the indexes related to foreleg power in striking, the Sanda players’ lead straight punching technique did not make good use of front leg start-up force. In training for cross-event athletes, lead leg active strength is the key for improving boxing abilities. In the selection of cross-event athletes for boxing, attention should be given to lower limb active strength indexes, such as lower limb CMJ and other biomechanical related indexes, during the evaluation of the lead straight punch.

## 5 Conclusion

The lead straight punching velocity, power, and other indexes should be evaluated when selecting athletes for the crossover from Sanda to boxing. The athletes with better lead straight punching effects should be selected. In both lower limbs power indexes, the lead leg start-up power index is a relatively important index for cross-event talent transfer and should not be ignored.

## Data Availability

The original contributions presented in the study are included in the article/Supplementary Material, further inquiries can be directed to the corresponding author.
